# A Novel Extended *N*-Methyl Monopyrrolotetrathiafulvalene Based on 2-Methylene-4,5-Bis(Methylthio)-1,3-Dithiole

**DOI:** 10.3390/molecules191220314

**Published:** 2014-12-04

**Authors:** Ruibin Hou, Xiaohong Shang, Yan Xia, Bao Li, Dongfeng Li

**Affiliations:** 1 School of Chemistry and Life Sciences, Changchun University of Technology, Changchun 130012, China; E-Mails: hrb1018@163.com (R.H.); shangxiaohong58@aliyun.com (X.S.); xiayan@mail.ccut.edu.cn (Y.X.); 2 Advanced Institute of Materials Science, Changchun University of Technology, Changchun 130012, China; 3 State Key Laboratory of Supramolecular Structure and Materials, Jilin University, Changchun 130012, China

**Keywords:** cross-coupling reaction, tetrathiafulvalene, pyrrole, crystal structure

## Abstract

The title compound was prepared via a cross-coupling reaction and its crystal structure has been determined. It crystallized in the triclinic space group *P*-1 with cell parameters: *a* = 8.552(2) Å, *b* = 11.310(2) Å, * c* = 16.150(3) Å, α = 109.55(3)°, β = 91.45(3)°, γ = 91.28(3)°, *V* = 1470.6(5) Å^3^, *Z* = 2 at 296 K. There is one molecule in the asymmetric unit. In the crystal structure, the neighboring molecules from dimers by weak intermolecular π···π interactions between the pyrrole and tetrathiafulvalene units. The dimers are further linked through C-H···π interactions to generate one-dimensional chains along the [100] direction. The arrangement of the molecules corresponds to an overlap between the HOMO and LUMO.

## 1. Introduction

**Figure 1:**
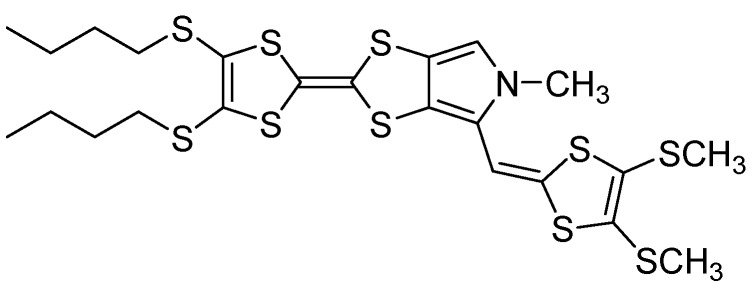
Extended MPTTF derivative **3**.

Tetrathiafulvalene (TTF) derivatives have played a pivotal role in the development of organic materials with optoelectronic applications because of their excellent electron-donating properties [1,2,3]. The TTF framework has been extensively modified over the past three decades to favor enhanced dimensionality in the related charge-transfer salts. Among those modifications, in heterocyclic-fused TTF donors a variety of molecules have been synthesized in which the TTF core is annelated to furan [4], dithiafulvene [5], thiophene [6] or selenophene [7] units. All of these compounds have oxidation potentials appreciably higher than that of TTF itself. Furthermore, there has been increased interest in incorporating the TTF moiety into supramolecular structures [8,9,10,11,12]. Becher and co-workers have presented a detailed study of pyrrolo-TTF and its *N*-alkylated derivatives [13]. The pyrrolo-annelated TTFs have extended π-surfaces, which can influence the physical properties of these heterocycles in both the solid state and in solution. Herein we report the molecular and crystal structure of a novel ex-TTF based on an electroactive monopyrrolo-TTF (**1**) and a 2-methylene-4,5-bis(methylthio)-1,3-dithiole (Figure 1). The crystal structure and electrochemical behavior are discussed.

## 2. Results and Discussion

### 2.1. Synthesis of Target Compound **3**

**Scheme 1:**
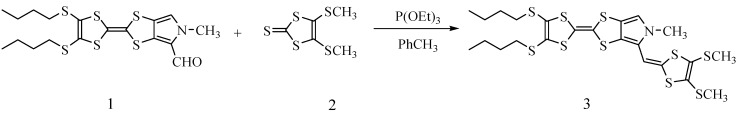
Synthesis of the extended MPTTF derivative **3**.

The novel extended MPTTF was synthesized as outlined in Scheme 1. Compound **1** was obtained according to reference [14]. The cross-coupling reaction of **1** with two equivalents of 4,5-bis(methyl-thio)-1,3-dithiole-2-thione (**2**) was carried out in toluene and triethyl phosphite at reflux to give the target molecule **3** in 36% yield [15]. It was characterized by ^1^H-NMR and ^13^C-NMR spectroscopy and mass spectrometry as listed in the Experimental section.

### 2.2. X-ray Structural Analysis of **3**

**Figure 2:**
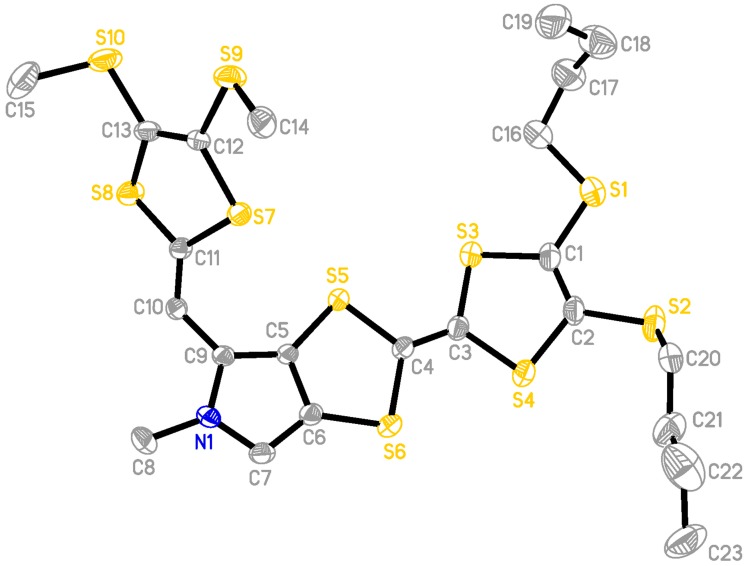
Molecular structure of compound **3**. Thermal ellipsoids are drawn at the 30% level. Hydrogen atoms are omitted for clarity.

**Figure 3:**
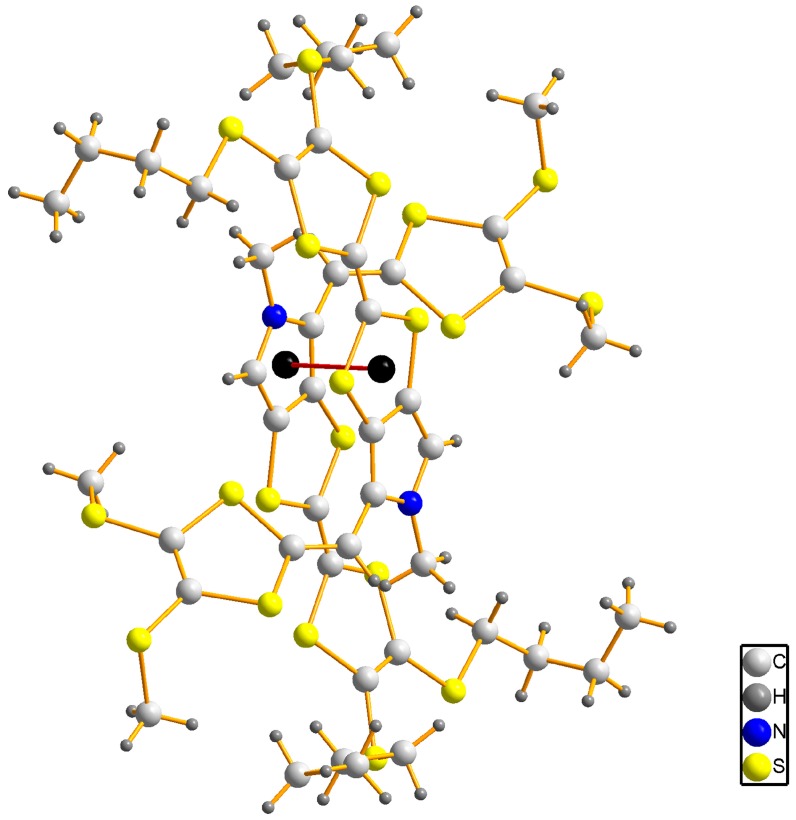
A ball-and-stick representation of compound **3**, showing the intermolecular π···π interactions in the dimer.

**Figure 4:**
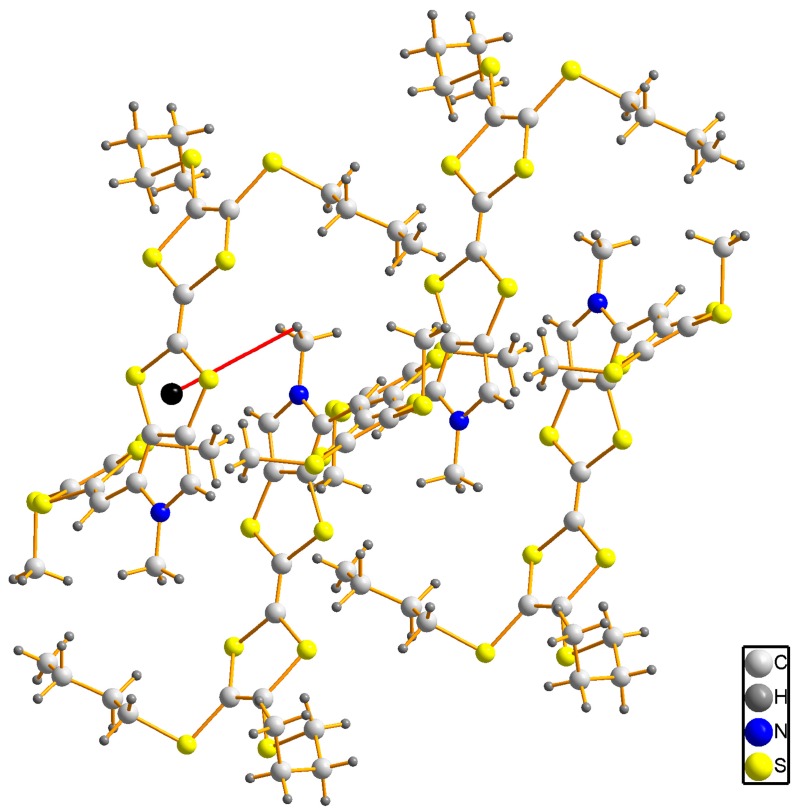
A ball-and-stick representation of compound **3**, indicating the intermolecular C-H···π interactions between two dimers.

**Figure 5:**
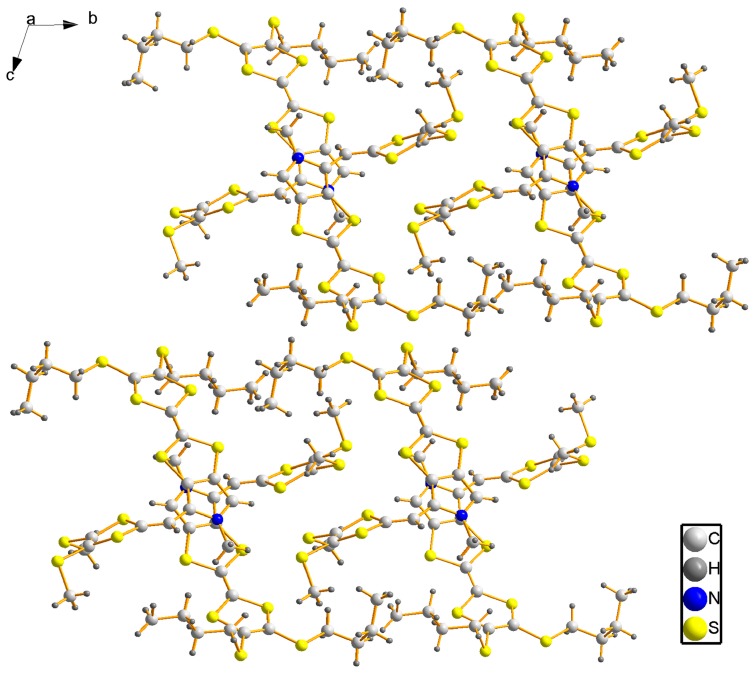
View of crystal packing of compound **3** along the *a* axis.

**Figure 6:**
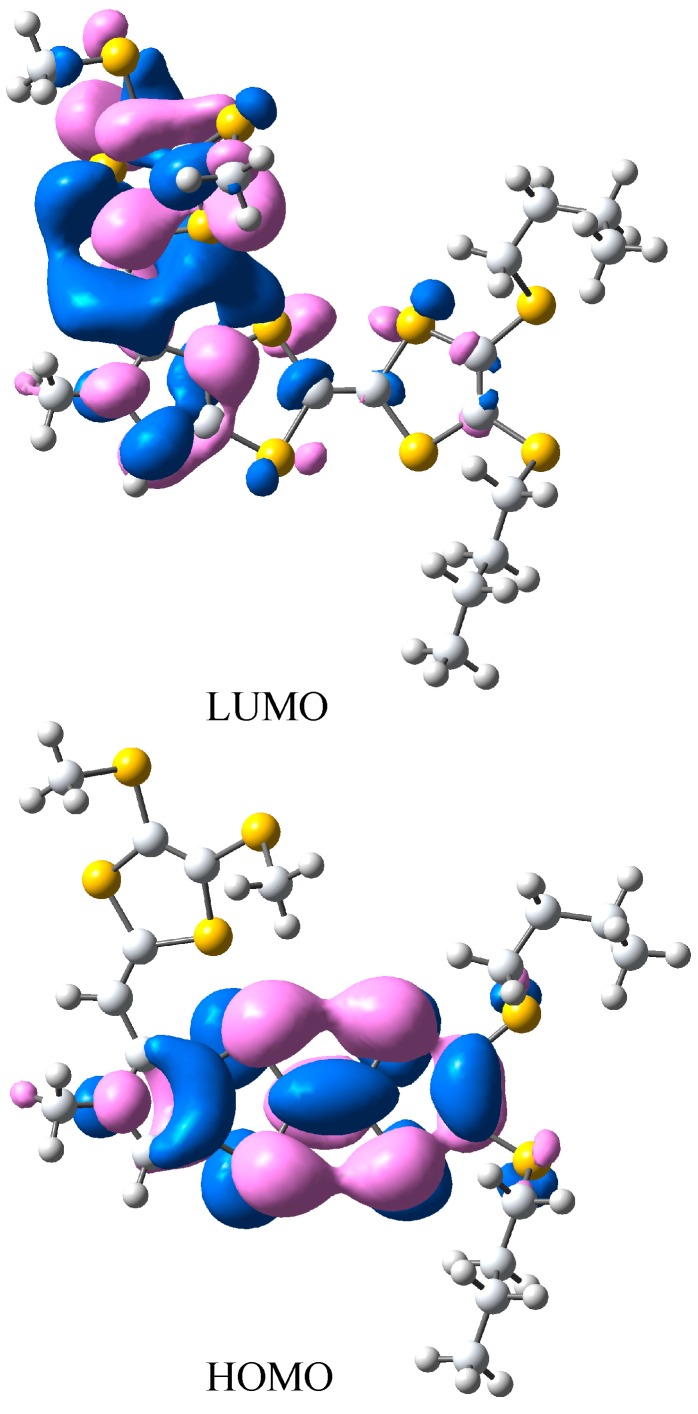
The HOMO and LUMO of compound **3**, calculated by single point B3LYP/6-31g(d) calculations.

The title compound **3** crystallizes in the triclinic space group *P*-1. As shown in Figure 2, there is one molecule in the asymmetric unit, and the bond lengths and angles are within normal ranges [16]. Two C atoms (C21 and C22) of one butyl exhibit disorder with occupancy ratio of 0.55:0.45. In the crystal structure, the pyrrolotetrathiafulvalene moiety and its attached methyl carbon atom (C8) lie on the same plane with an r.m.s. deviation of 0.0278 Å. At either end of the pyrrolotetrathiafulvalene ring, there are two TTF moieties with different alkyl chains which have a similar configuration. The first TTF moiety has two butyl groups, and the TTF ring and the neighboring two S atoms (formed by S1 to S4 and C1 to C3) can be divided into three parts based on their planarity: the dihedral angle between plane 1 (averaged from C1, C2, S1 and S2) and plane 2 (averaged from C1, C2, S3 and S4) is 5.3(3)°, which shows they are almost coplanar. The angle between between plane 2 and plane 3 (formed from C3, S3 and S4) however, is 22.6(4)°, which is an obvious deviation from co-planarity. The second TTF moiety with methyl groups possesses the similar conformation with the dihedral angels of 6.1(3)° (between the averaged planes of C12, C13, S9, S10 and C12, C13, S7, S8) and 19.4(4)° (between the averaged planes of C12, C13, S7, S8 and C11, S7, S8). These configurations of TTF moieties are similar to previous reports [17]. In the crystal structure of compound **3**, the weak intermolecular π-π interactions between pyrrole and TTF of neighboring molecules form a dimer with a centroid to centroid distance of 3.743(2) Å, as shown in Figure 3. The dimers are further linked through the intermolecular C-H···π interactions with a hydrogen to centroid distance of 3.873(1) Å into one-dimensional chains along [100] direction, as shown in Figure 4. The packing view of compound **3** shows a sheet structure, as is shown in Figure 5. The arrangement of the molecules corresponds to the overlap of HOMO and LUMO for **3**, as shown in Figure 6.

### 2.3. Electrochemistry

**Figure 7:**
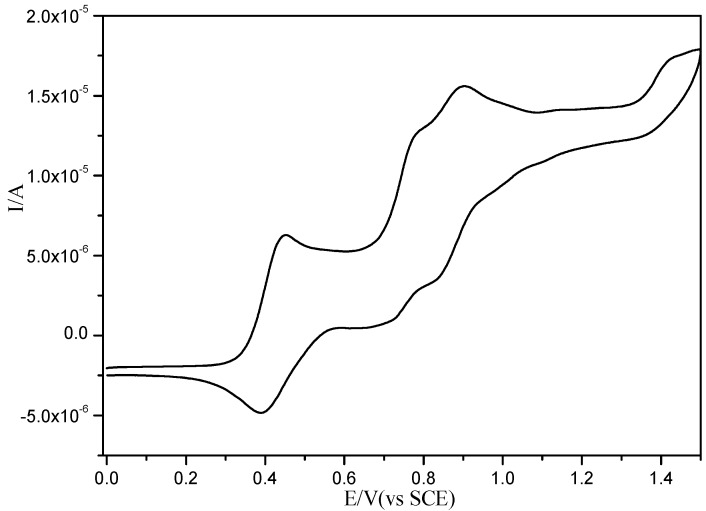
Cyclic voltammograms (CV) of compound **3** in CH_2_Cl_2_/CH_3_CN (4:1, v/v) containing 0.1 M Bu_4_PF_6_.

The electrochemical characterisation of the compound **3** was carried out using cyclic voltammetry under standard conditions. For comparison purposes we also investigated the electrochemical behaviour of the formyl-MPTTF **1** under similar conditions. The results were listed in Table 1. It showed two reversible, monoelectronic oxidation processes, which were associated with the successive oxidation of the TTF unit to TTF^+^ and TTF^2+^, respectively [15]. In contrast, CV analysis of **3** displayed a complicated cyclic voltammogram as shown in Figure 7, the first process was fully reversible and corresponded to the concomitant oxidation of both neutral MPTTF into the cation radical species while the second process was not fully reversible and showed split second redox couples, with E_1/2_^2^ at 0.75 V and 0869 V, which probably was caused by adsorption phenomena on the electrode while the third process was fully irreversible in the positive direction and the listed oxidation potentials were only approximate, with E_ox_^3^ at ~1.422 V [5,18,19].

**Table 1:** Half-wave (E_1/2_) and Oxidation (E_ox_) potentials for compounds **1**, **3**.

Compound	E_1/2_^1^ (V)	E_1/2_^2^ (V)	E_ox_^3^ (V)
**1**	0.470	0.773	-
**3**	0.422	0.869 (0.750)	1.423

^1^ The first half wave potential; ^2^ The second half wave potential; ^3^ The third oxidation potential.

### 2.4. Electronic Absorption Spectroscopy

**Figure 8:**
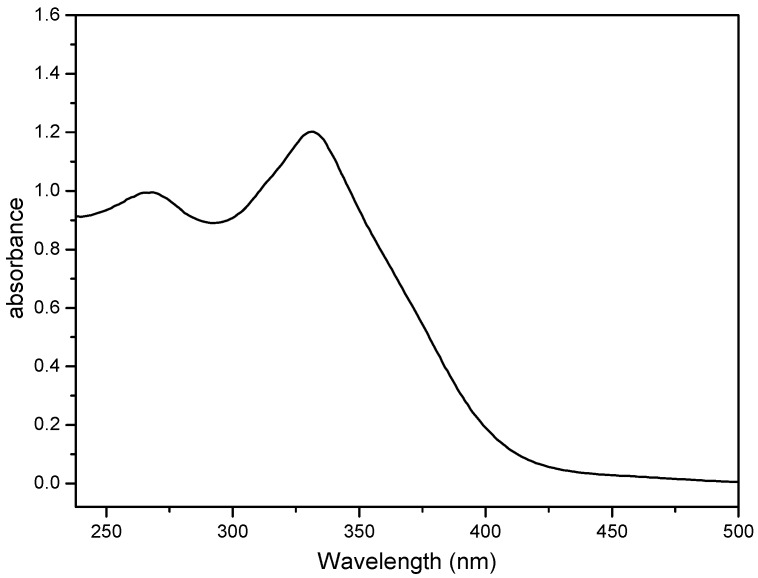
UV–Vis spectrum of compound **3** recorded in CH_2_Cl_2_ (4 × 10^−5^ mol/L) at room temperature.

The photophysical properties have been studied in air-equilibrated CH_2_Cl_2_ solutions at room temperature. The electronic UV–Vis spectrum for **3** is displayed in Figure 8. It transpires that compound **3** and exhibit very broad absorption bands at 269 nm (ε 22849 M^−1^·cm^−1^) and 331 nm (ε 28817·M^−1^·cm^−1^), respectively.

## 3. Experimental Section

### 3.1. General Information

All solvents were purified or dried by standard methods. All reagents used were obtained from ACROS (Geel, Belgium) and used without purification. The silica gel used was 100–200 mesh from Qing Dao Hai Yang Chemical Factory (Qing Dao, china). NMR spectra were recorded in CDCl_3_ with an AV-300 spectrometer Bruker (Bruker, Karlsruhe, Germany). Chemical shifts are given relative to tetramethylsilane (δ_H_/δ_C_ = 0 ppm). MALDI-TOF MS data were obtained with an AXIMA-CFR^TM^ plus mass spectrometer (Shimadzu, Tokyo, Japan) using a 1,8,9-anthracenetriol (DITH) matrix. The crystal data were measured with a SCX mini-diffractometer (Rigaku, Tokyo, Japan) with Mo-Kα radiation (λ = 0.71073 Å) in ω scan mode at 296(2) K. Cyclic voltammetric studies were carried out on a 273 A Potentiostat/Galvanostat instrument (company, Potsdam, Germany) in CH_2_Cl_2_/CH_3_CN (4:1, *c* = 1 × 10^−3 M) and 0.1 MBu4PF6 as the supporting electrolyte and scan rate is 20 mV·S−1^. Counter and Working electrodes were made of Pt and Glass-Carbon, respectively, and the reference electrode was calomel electrode (SCE). Compound **1** was synthesized according to literature methods [14].

### 3.2. Synthesis of N-Methyl-4-[4,5-Bis(Metylthio)-l,3-Dithiol-2-Yliden]-2-[4,5-Bis-(1-Butyithio)-l,3-Dithio1-2-Yli-Den]-(1,3)-Dithiolo[4,5-c]Pyrrole (**3**)

To solution of **1** (0.0752 g, 0.1629 mmol) and 4,5-bis(methylthio)-1,3-dithiole-2-thione (0.0737g, 0.3257 mmol) in dry toluene (15 mL) was added P(OEt)_3_ (2 mL) under Ar. The reaction mixture was stirred 5 h at 120 °C. The solvent was evaporated under vacuum. The solid was purified by column chromatography (silica gel, CH_2_C1_2_/PE = 1:2, R_f_ = 0.2) to give **3**. Recrystallization from CH_2_C1_2_/PE gave 0.0375 g yellow-brown needles (36%). mp 112–113 °C, ^1^H-NMR(CDCl_3_/TMS): δ 0.94 (t, *J* = 7.3 Hz, 6 H), 1.41–1.49 (m, 4 H), 1.56–1.66 (m, 4 H), 2.44 (s, 3 H), 2.46 (s, 3 H), 2.83 (t, *J* = 7.2 Hz, 4 H), 3.50 (s, 3 H), 6.17(s, 1 H), 6.46(s, 1 H); ^13^C-NMR(CDCl_3_): 13.57, 18.93, 21.61, 31.75, 35.12, 35.90, 110.39, 118.06, 122.96, 125.22, 126.98, 127.09, 127.55; MALDI-TOF-MS, found: *m/z* 638.96.

### 3.3. X-ray Crystallography

Diffraction data were collected on a *RAPID-AUTO* diffractometer fitted with a CCD type area detector, and a full sphere of data were collected using graphite-monochromated Mo Kα radiation (λ = 0.71073 Å). The data frames were integrated using SAINT and merged to give a unique data set for structure determination. The structures were solved by direct methods and refined by the full-matrix least-squares method on all F^2^ data using the SHELEX program (Bruker Analytical X-ray System). Empirical absorption corrections by SADABS were carried out. Non-hydrogen atoms were refined with anisotropic thermal parameters. Hydrogen atoms were included in calculated positions and refined with isotropic thermal parameters riding on those of the parent atoms. Crystal data and refinement details are summarized in Table 2.

**Table 2:** Crystal data and refinement details for **3**.

Chemical Formula	C23 H29 N S10
Formula weight	640.07
Temperature	296(2) K
Wavelength	0.71070 Å
Crystal system	Triclinic
Space group	P-1
a (Å)	8.552(2)
b (Å)	11.310(2)
c (Å)	16.150(3)
α(°)	109.55(3)°
β(°)	91.45(3)°
γ(°)	91.28(3)°
V (Å^3^)	1470.6(5)
Z	2
Calculated density	1.445 Mg·m^−3^
Absorption coefficient	0.77 mm^−1^
F(000)	668
Crystal size	0.39 mm × 0.25 mm × 0.06 mm
θ Range for data collection	3.01–27.55
Index ranges	−11 ≤ h ≤ 11
	−14 ≤ k ≤ 14
	−20 ≤ l ≤ 20
Completeness to θ	98.5%
Reflections collected	14,464
Independent reflections	6633 [Rint = 0.067]
Absorption correction	None
Refinement method	Full-matrix least-squares on F^2^
Data/restrains/parameters	6633/6/345
Goodness-of-fit on F^2^	1.024
Final R indices [I > 2σ(I)]	R_1_ = 0.0642, wR2 = 0.1278
R indices (all data)	R_1_ = 0.1379, wR2 = 0.1601
Largest diff. peak and hole Å^−3^	0.41 and −0.38 eÅ^−3^

CCDC 978639 contains the supplementary crystallographic data for this paper. These data can be obtained free of charge via website [20], by e-mailing data_request@ccdc.cam.ac.uk or by contacting The Cambridge Crystallographic Data Centre, 12, Union Road, Cambridge CB2 1EZ, UK; fax: +44-1223-336033.

### 3.4. Theoretical Calculations

The molecular orbital shapes of HOMO and LUMO of compound **3** were evaluated in single point 6-31G(d) [21] calculations using the crystallographic geometry of **3** with Gaussian 09 [22].

## 4. Conclusions

We have determined the crystal structure of an extended monopyrrolotetrathiafulvalene derivative **3** by X-ray diffraction. Compound **3** crystallized in the triclinic space group *P*-1 with one molecule in the asymmetric unit. The packing view of **3** shows it adopts a sheet structure. The dimers, linked between pyrrole and TTF of neighboring molecule by π-π interactions, are connected into 1D chains along the [100] direction by C-H···π interactions.
